# Biomarker interaction selection and disease detection based on multivariate gain ratio

**DOI:** 10.1186/s12859-022-04699-7

**Published:** 2022-05-12

**Authors:** Xiao Chu, Mao Jiang, Zhuo-Jun Liu

**Affiliations:** 1 Academy of Mathematics and Systems Science Chinese Academy of Sciences, University of Chinese Academy of Sciences, Beijing, China; 2 Academy of Mathematics and Systems Science Chinese Academy of Sciences, Beijing, China

**Keywords:** Multivariate gain ratio, Biomarker interaction, Disease detection

## Abstract

**Background:**

Disease detection is an important aspect of biotherapy. With the development of biotechnology and computer technology, there are many methods to detect disease based on single biomarker. However, biomarker does not influence disease alone in some cases. It’s the interaction between biomarkers that determines disease status. The existing influence measure *I*-score is used to evaluate the importance of interaction in determining disease status, but there is a deviation about the number of variables in interaction when applying *I*-score. To solve the problem, we propose a new influence measure Multivariate Gain Ratio (MGR) based on Gain Ratio (GR) of single-variate, which provides us with multivariate combination called interaction.

**Results:**

We propose a preprocessing verification algorithm based on partial predictor variables to select an appropriate preprocessing method. In this paper, an algorithm for selecting key interactions of biomarkers and applying key interactions to construct a disease detection model is provided. MGR is more credible than *I*-score in the case of interaction containing small number of variables. Our method behaves better with average accuracy $93.13\%$ than *I*-score of $91.73\%$ in Breast Cancer Wisconsin (Diagnostic) Dataset. Compared to the classification results $89.80\%$ based on all predictor variables, MGR identifies the true main biomarkers and realizes the dimension reduction. In Leukemia Dataset, the experiment results show the effectiveness of MGR with the accuracy of $97.32\%$ compared to *I*-score with accuracy $89.11\%$. The results can be explained by the nature of MGR and *I*-score mentioned above because every key interaction contains a small number of variables in Leukemia Dataset.

**Conclusions:**

MGR is effective for selecting important biomarkers and biomarker interactions even in high-dimension feature space in which the interaction could contain more than two biomarkers. The prediction ability of interactions selected by MGR is better than *I*-score in the case of interaction containing small number of variables. MGR is generally applicable to various types of biomarker datasets including cell nuclei, gene, SNPs and protein datasets.

## Background

Disease detection via biomarkers (e.g. genes) is an indispensable part of medical research. In the last couple of years, remarkable progress has been made in the field via connecting the biomarkers and outcome variable. Most such studies have used a single-biomarker analysis strategy for selecting every important predictor variable individually. Nowadays, a growing body of evidence shows that the interaction between biomarkers is the cause of disease [1–5]. If a biomarker plays an important role in disease detection when combined with other biomarkers, it will be missed by using the single-biomarker selection method. There are reviews of using the interaction between biomarkers to detect disease [6–9].

Discovering important biomarkers and their interactions that account for disease status has continued to be a key challenge in biotherapy expression analysis [10]. The dataset is often with tens or hundreds of samples and thousands or even more biomarkers. We take biomarker interactions into consideration in reality, which makes the problem of selecting biomarker interactions more serious [11]. The traditional statistical tests are less useful and it is particularly crucial to select key biomarkers and their interactions for effective data analysis.

In order to select the interaction of biomarkers, an effective influence measure *I*-score was proposed by Shaw-Hwa Lo [12]. There are several advantages of *I*-score in biomarker interactions’ detection. But there still exists a shortcoming because *I*-score is a biased measure about the number of variables in interaction. The deviation of *I*-score can’t satisfy the premise of backward dropping algorithm (BDA).

To solve the problem of *I*-score, we propose a new influence measure Multivariate Gain Ratio (MGR) based on single-variate Gain Ratio (GR) in information theory. There are many studies about using information theory to detect interactions between biomarkers [13–17]. However, these methods can’t deal with the problem of high-dimension interactions among biomarkers. MGR overcomes the problem of high-dimension interactions. It can select the important biomarker interactions based on the information they provide for the determination of outcome variable. In addition, MGR maintains the advantage of *I*-score: it does not require one to specify a model for the joint effect of biomarkers on outcome variable.

We carry out some experiments to analyze characteristics of the two influence measures *I*-score and MGR. MGR behaves better in the case of interactions with small number of variables. In addition to detecting important interactions, another objective of this paper is to predict the patients’ disease status based on the selected biomarkers and their interactions. We choose Ridge Regression method with cross-validation to make it. The classification results show the effectiveness of biomarkers and their interactions found by MGR in determining disease. Finally, we get the conclusion that MGR is better than *I*-score when there is small number of biomarkers in key interaction.

As we all know, preprocessing is an important aspect of machine learning. We need to choose an appropriate preprocessing method before finding interactions in biotherapy. Therefore, we propose a preprocessing verification algorithm based on partial predictor variables to select an appropriate preprocessing method. An integrated structure for selecting important biomarker interactions in biotherapy and applying interactions to detect disease is provided in this paper.

The rest of the paper is organized as follows. In the methods section, we introduce the influence measures *I*-score and MGR. In order to explore the nature of the two measures, we perform some random experiments. In addition, we show the algorithm of selecting important biomarker interactions and constructing classifiers for outcome variable thoroughly. As a result, we show the effectiveness of MGR by carrying out experiments on two real datasets. Finally, the conclusion and discussion of the paper including future research directions will be stated.

## Dataset

The first dataset to be considered is Breast Cancer Wisconsin (Diagnostic) Dataset, which is available at UCI Machine Learning Repository [18]. The problem is to distinguish malignant (cancerous) from benign (non-cancerous) examples. The data has 569 examples, 357 benign and 212 malignant. There are 30 features for every sample. Features are computed from a digitized image of a fine needle aspirate (FNA) of a breast mass. The features describe the characteristics of the cell nuclei present in the image [19].

The second dataset is Leukemia Dataset [20]. This dataset comes from a study of gene expression in two types of acute leukemias including acute lymphoblastic leukemia (ALL) and acute myeloid leukemia (AML). There are 72 samples totally including 47 cases of ALL and 25 cases of AML. Gene expression levels were measured using Affymetrix high-density oligonucleotide arrays. Every sample contains $p=7129$ human gene expression value. The dataset is available at [21]. After three preprocessing procedures applied to the normalized matrix of intensity values [22], the number of genes is reduced to 3571 The preprocessing details could also be seen in section results.

## Methods

### Comparison and analysis of *I*-score and MGR

Suppose that there is a design matrix *X* with dimension of $n\times p$, where *n* is the number of samples and *p* is the number of predictor variables $\left\{ X_1,X_2,\ldots ,X_p\right\}$. In addition, every sample has an outcome variable *Y*. So it is of interest to identify main predictor variables as well as their interactions based on the design matrix and outcome variable. Here we make use of two influence measures including the existing *I*-score and our proposed MGR separately to assess the importance of predictor variables and their interactions. Without losing generality, we assume that the outcome variable *Y* is binary with value 0 and 1. At the same time, all predictor variables are discrete. Consider the partition $P_k$ generated by a subset of *k* predictor variables $S_b=\left\{ X_{b_1},\ldots ,X_{b_k} \right\}$, $1\le b_1\le b_2\le \cdots \le b_k\cdots \le p$. If all predictor variables in the subset are binary then there are $2^k$ partition elements in partition $P_k$. Let $n_1(j)$ be the number of observations with $Y=1$ in partition element *j* where $j\in P_k$. Let ${\bar{n}}_1(j)=n_j\times \pi _1$ be the expected number of $Y=1$ in element *j* under the null hypothesis that the subset of predictor variables has no association with *Y*, where $n_j$ is the total number of observations in element *j* and $\pi _1$ is the proportion of $Y=1$ observations in the sample. The *I*-score of $\left\{ X_{b_1},\ldots ,X_{b_k}\right\}$ is defined as [23]

1 $${I}(S_b)=\sum _{j\in P_k}\left[ n_1(j)-{\bar{n}}_1(j)\right] ^{2}.$$

Gain Ratio with one-dimension predictor variable *X* is as follows [24]:

2 $$\mathrm{GR}(X)=\frac{Gain(X)}{SplitInfo(X)}=\frac{Info(Y)-Info _X(Y)}{SplitInfo(X)},$$

where *Gain*(*X*) is the information gain provided by the predictor variable *X* in determining the outcome variable *Y*, *SplitInfo*(*X*) is the self-information of predictor variable *X*. Because the predictor variables’ classification number affects the results, Quinlan uses the self-information of predictor variable to divide information gain to correct the deviation of variables’ classification number [24]. Combined with the above hypothetical scenarios, we extend the one-dimension Gain Ratio to the multi-dimension case, which is Multivariate Gain Ratio:

3 $$\begin{aligned} \mathrm{MGR}(S_b)&=\frac{Gain(S_b)}{SplitInfo(S_b)}\\&=\frac{Info(Y)-Info _{S_b}(Y)}{SplitInfo(S_b)}, \end{aligned}$$

where $$Info(Y)=-\pi _1log(\pi _1)-(1-\pi _1)log(1-\pi _1)$$,

$$Info _{S_b}(Y)=-\sum _{j\in P_k}(\frac{n_1(j)}{n}log(\frac{n_1(j)}{n_j})+\frac{n_j-n_1(j)}{n}log(\frac{n_j-n_1(j)}{n_j})),$$

$$SplitInfo(S_b)=-\sum _{j\in P_k}\frac{n_j}{n}log(\frac{n_j}{n})$$.

*Info*(*Y*) measures the average amount of information needed to identify the outcome variable of a case. $Info _{S_b}(Y)$ is the needed average information to identify the outcome variable of a case when the outcome variable *Y* is partitioned in accordance with the subset $S_b$. Then $Gain(S_b)$ measures the information gained by partitioning outcome variable *Y* in accordance with subset $S_b$. $SplitInfo(S_b)$ is the self-information of predictor variables $S_b$, which represents the potential information generated by dividing the samples into $2^k$ subsets. Because $Gain(S_b)$ has a strong bias in favor of $S_b$ with many classification numbers, we need to use $SplitInfo(S_b)$ as a kind of normalization to rectify $Gain(S_b)$. Then, $\mathrm{MGR}(S_b)$ expresses the proportion of information generated by the split and useful for classification. GR is an influence measure which could be used to detect important single biomarker variable. However, if a biomarker plays an important role in disease detection when combined with other biomarkers, it will be missed by using GR method. The following simulated example could illustrate the advantages of MGR in determining biomarker interactions. We generate a predictor variable matrix $X_{200\times 10}$ by randomly uniformly sampling from $\left\{ 0,1\right\}$, where 200 is the number of samples and 10 is the number of predictor variables. The outcome variable *Y* is related to $X_{200\times 10}$ via the model

$$\begin{aligned} Y=\left\{ \begin{aligned} X_1(module2)\; with\; probability\; 0.5\\ X_2+X_3(module2)\; with\; probability\; 0.5 \;. \end{aligned} \right. \end{aligned}$$

**Fig. 1:**
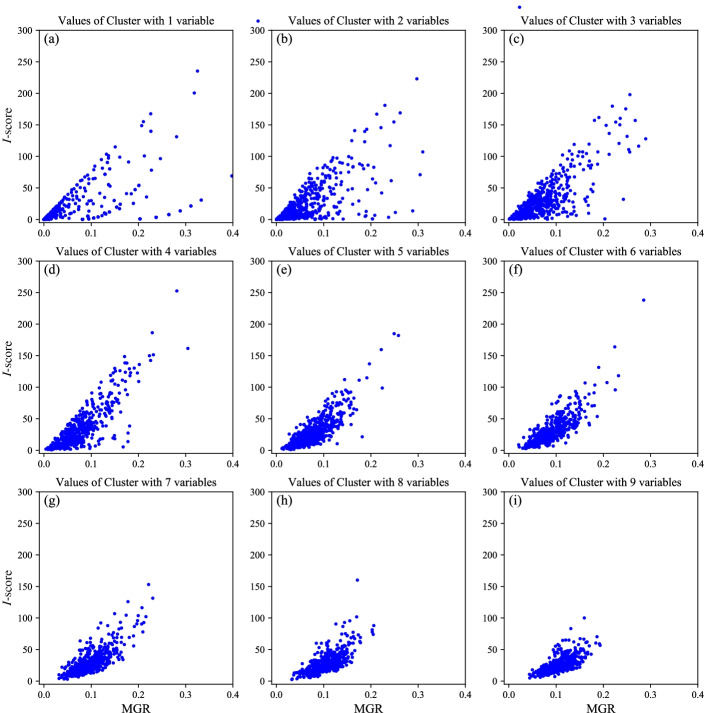
Scatter plots of correlation between *I*-score and MGR. For example, when $p=1$, we sample one variable as an interaction from 3571 predictor variables 500 times. We document *I*-score and MGR of the interaction as a numerical pair. Then we get the scatter plot Values of Cluster with 1 variable in **a**. *I*-score and MGR are consistent in the nature of growth

Table 1 is the GR value of ten predictor variables from high to low. GR couldn’t select important single variable such as $X_2$. However, from the simulated setting, $X_2$ is an important variable in determining *Y* when combined with $X_3$. The MGR of interaction $\left\{ {X_2,X_3} \right\}$ is $84.4\times 10^{-3}$, which illustrates that $\left\{ {X_2,X_3} \right\}$ could be detected by MGR. MGR does not require one to specify a model for the joint effect of $\left\{ {X_{b_1},\ldots ,X_{b_k}} \right\}$ on *Y*. It is designed to capture the information provided by $\left\{ {X_{b_1},\ldots ,X_{b_k}} \right\}$ for determining *Y*. The property makes MGR a useful measure. We carry out two experiments in order to explore the nature of *I*-score and MGR. In the first experiment, we use Leukemia Dataset [20] to explore if there are something in common between *I*-score and MGR without loss of generality. We repeat sampling *p* variable(s) from 3571 predictor variables 500 times ($p=1, 2,\ldots ,9$). For example, when $p=1$, we sample one variable as an interaction from 3571 predictor variables 500 times. We document *I*-score and MGR of the interaction as a numerical pair. Then we get the scatter plot Values of Cluster with 1 variable as Fig. 1a.

**Table 1:** GR value of ten predictor variables

Predictor variable	$X_1$	$X_6$	$X_3$	$X_{10}$	$X_8$	$X_7$	$X_2$	$X_9$	$X_4$	$X_5$
GR ($\times 10^{-3}$)	250.4	16.3	8.8	7.2	7.2	6.1	5.9	1.2	0.7	0.7

**Fig. 2:**
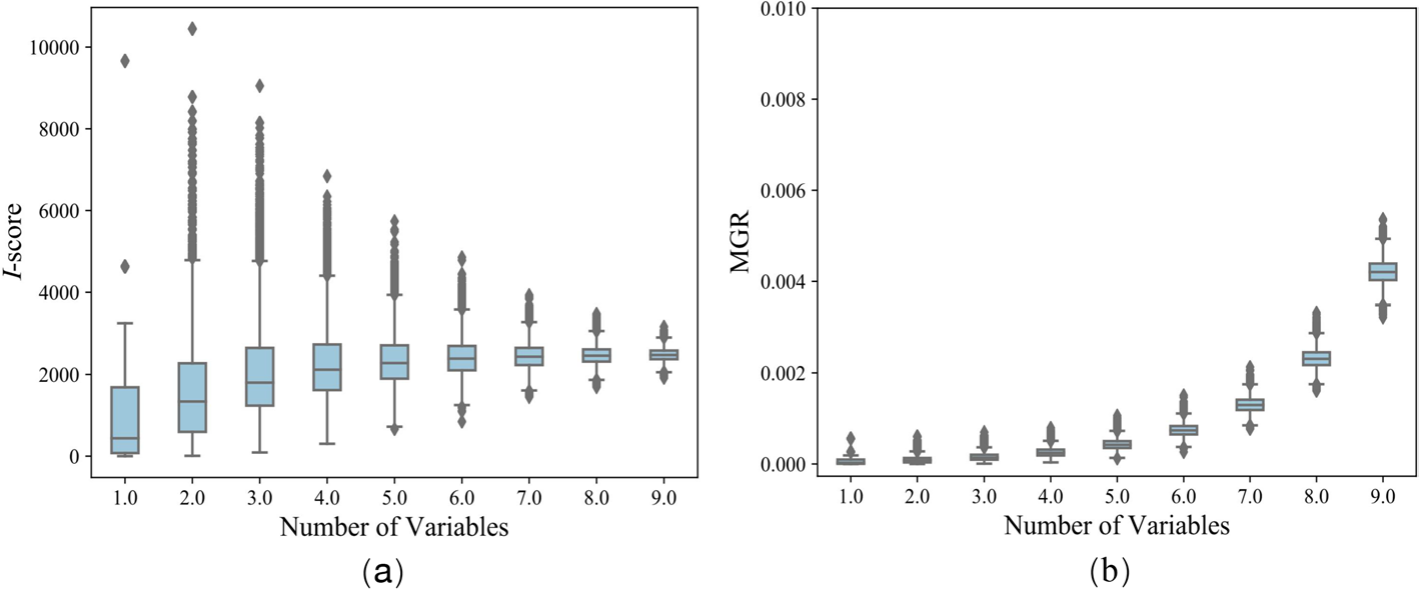
Variation of *I*-score and MGR with variable number in range 1 and 9. **a** *I*-score varies with the number of variables in logarithmic function form, while MGR is in the form of the exponential function as shown in **b**

Due to the experiment results, *I*-score and MGR are consistent in the nature of growth. Next, we are ready to explore the difference between the two influence measures. According to Fig. 1a–i, the correlation of *I*-score and MGR varies with the number of variables in interaction. If we want to compare the importance of interactions with different number of variables, the influence measure must meet the demand that it’s unbiased for interactions with different number of variables. Then the value of influence measure stands for the importance of interactions completely rather than the interference of the number of variables. We continue to find out the law that *I*-score and MGR separately vary with the number of variables in interaction. Every time we randomly generate interaction containing *p* predictor variable(s) and outcome variable *Y*, $p=1,2,\ldots ,9$. Under this setting, outcome variable is independent of every predictor variable interaction. For fixed variable number *p*, we generate a predictor variable matrix $X_{10{,}000\times p}$ with dimension $10{,}000\times p$ by randomly uniformly sampling from $\left\{ 0,1\right\}$ and outcome variable with dimension $10{,}000\times 1$ by randomly uniformly sampling from $\left\{ 0,1\right\}$. Then record the corresponding value *I*-score and MGR of the interaction. Finally, we repeat the whole process 20,000 times. We draw the boxplot among the 20,000 interactions for every fixed *p*. Finally we get the results that the two influence measures vary with number of variables separately shown as Fig. 2.

We could explore the trend of *I*-score and MGR varying with the number of variables in interaction by the boxplot Fig. 2. Because every predictor variable and outcome variable are generated randomly and independently, the interaction with different predictor variables should be independent of outcome variable. Therefore, the two influence measures should not significantly vary with the number of predictor variables in interaction, which is the basis of using the two influence measures to evaluate the importance of interaction with different number of variables. However, Fig. 2 shows that the two influence measures both increase along with the number of variables. But the specific growth situation of them is different. When the number of variables is between 1 and 9, Fig. 2a shows that *I*-score varies with the number of variables in logarithmic function form, while MGR is in the form of the exponential function as shown in Fig. 2b. With the increase of variables, *I*-score increases rapidly, then the rate of increase slows down, and finally the values tend to be stable. While the increasing performance of MGR is opposite to that of *I*-score. When there is key interaction with a relatively small number of predictor variables in the datasets, the selected key interaction based on *I*-score contains other unimportant variables while carrying out BDA. Therefore we can come to a conclusion that MGR performs better in the case of interaction with a relatively small number of variables, and *I*-score is more suitable for interaction with more variables. Based on the diagram, the results of MGR are more credible than *I*-score when the number of predictor variables in interaction is less than five.

### Algorithms

In the first part of this section, we introduce BDA which is a repeated sampling algorithm used to find out the interactions. BDA is a greedy algorithm aiming at iterating over all the variables. Every time an initial predictor variable subset is selected, then delete variables one by one and finally find out the subset with maximum influence measure. The details are as follows.

1. Training set: Consider a training set ${(y_1,x_1),\ldots ,(y_n,x_n)}$, where $x_i=(x_{1i},\ldots ,x_{pi})$ with the dimension of *p* and the number of samples *n*. Typically *p* is very large. Outcome variable and all predictor variables are discrete.
2. Sampling for initial subset: Select *k* predictor variables $S_b=\left\{ X_{b_1},\ldots ,X_{b_k} \right\}$, $b=1,\ldots ,B$ as initial subset. *B* is the number of repetitions which will be introduced in step 3.
3. Compute the influence measure of the initial subset.
4. Drop variables: Tentatively drop each variable in $S_b$ in turn and recalculate the influence measure of the subset with one variable less. Then drop the one that gives the highest influence measure. Keep this retained pending combination as $S'_b$.
5. Return set: Continue the next round of dropping on $S'_b$, until only one variable is left. Now we have *k* retained pending combinations with variable numbers ranging from *k* to one. Keep the subset that yields the highest influence measure among the retained pending combinations. Refer to this subset as the return set $R_b$. Keep it for future use.

**Fig. 3:**
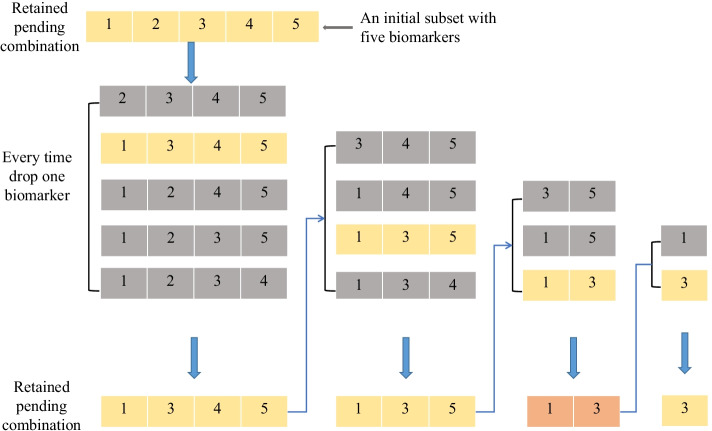
Illustration of BDA. In this example, we randomly select five biomarkers $\left\{ x_{b_1},x_{b_2},x_{b_3},x_{b_4},x_{b_5}\right\}$ as the initial subset. For ease of display, the biomarkers $\left\{ x_{b_1},x_{b_2},x_{b_3},x_{b_4},x_{b_5}\right\}$ is represented by the subscript interaction $\left\{ 1,2,3,4,5\right\}$

**Fig. 4:**
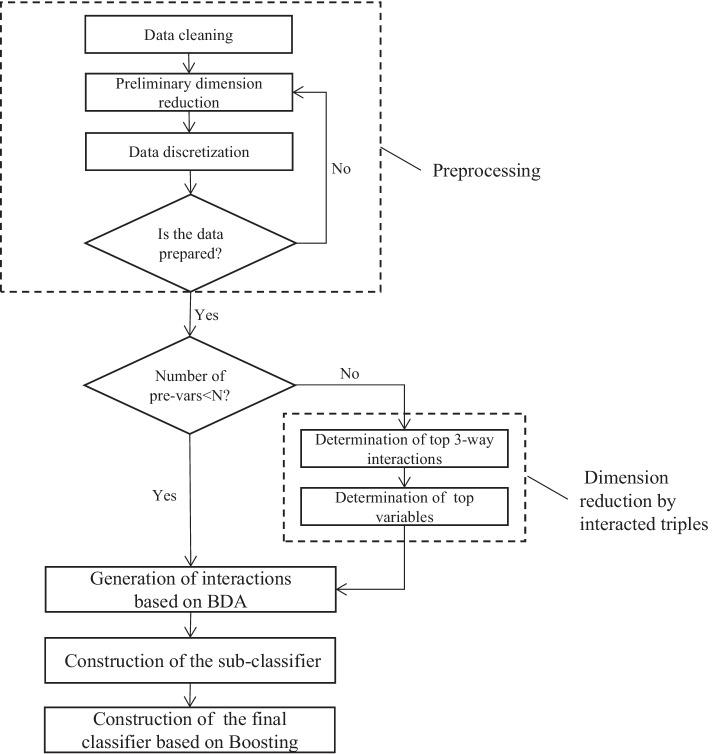
Flowchart of proposed interaction selection and classifier construction. There are five main steps in the algorithm including preprocessing, dimension reduction by interacted triples, generation of interactions based on BDA, construction of the sub-classifier, construction of the final classifier based on Boosting

For ease of understanding, we construct an example as shown in Fig. 3 to illustrate how BDA is conducted in detail. In this example, we tentatively drop one biomarker from $\left\{ 1,2,3,4,5\right\}$ in turn, and document the influence measure of the interaction with four remaining biomarkers. The interaction with the largest influence measure is put into the retained pending combination pool. Then drop every biomarker of it. Repeat the above process until only one biomarker is left. Finally we choose the retained pending combination with the largest influence measure as the return set. When the number of predictor variables is small enough, we can obviously use the BDA directly with the influence measure such as *I*-score or MGR to find interactions. If the predictor variables we meet are in tens of thousands, we must do some preparation before using BDA. We propose an algorithm to select important interactions in detail and construct the classifier of disease detection. The algorithm consists of five steps as shown in Fig. 4.

#### Step 1: Preprocessing

With the increase of the amount of biological data, searching for more effective computing algorithms will become an indispensable goal. Therefore, we need to apply preprocessing algorithm to help us preliminarily select valuable predictor variables. And we propose a preprocessing method in this paper, which could save the time of finding the appropriate preprocessing method. Preprocessing mainly includes four sub-steps: data cleaning; preliminary dimension reduction; data discretization; judgement of candidate method.

1. Data cleaning: The original dataset is transformed to $n\times p$ dimension, in which *n* stands for the number of samples, and *p* is the number of predictor variables. The sample status also needs to be prepared, which is represented by variable *Y*.
2. Preliminary dimension reduction: In this sub-step, we need to make sure if every predictor variable is meaningful in determination of variable *Y*. There are many methods such as t-test and Fold-change [25] aiming at selecting variables preliminarily. We choose the appropriate method to make it under different situations. We filter the biomarkers preliminarily, which is beneficial for our program later.
3. Data discretization: There are many discretization methods such as equal width method, equal frequency method, clustering method [26]. The most appropriate discretization method varies through different backgrounds.
4. Judgement of candidate method: We consult literature based on the background of the dataset to identify candidate methods about preliminary dimension reduction and data discretization. Then do some pre-experiments to determine the best candidate. For example, after dimension reduction and discretization, we carry out important interaction extraction experiments by BDA based on randomly chosen one hundred predictor variables with discrete values. If the accuracy of the classifier constructed by step 4 and step 5 based on these selected important interactions is reasonable, we carry out experiments on the whole dataset with the candidate preprocessing method.

Our proposed preprocessing method saves computation time and improves efficiency which is benefit for step 2 and step 3. If the number of predictor variables in the dataset is small enough, then step 2 can be omitted to get the important interactions directly with BDA.

#### Step 2: Dimension reduction by interacted triples

When the number of predictor variables is in the thousands or even larger after preprocessing, we must screen the predictor variables before applying BDA. We take interaction effect into consideration in the process of screening. We realize the dimension reduction by 3-way interactions (triples) [23]. This step consists of two sub-steps including obtaining top 3-way interactions and top variables.

1. Determination of top 3-way interactions: Firstly, sort the triples from high to low according to their respective influence measure. Then pick a triple out every 1000 triples from the beginning of the ordered sequence. Typically, the second difference of sequence consisting of every thousandth triple’s scores declines sharply in the beginning and stabilizes around zero later. We will choose top 3-way interactions according to the second difference scores of thousandth near to zero for the first time. And the top thousands of triples are retained for further analysis.
2. Determination of top variables: After the above sub-step, each variable in the high-scored triples may occur in multiple triples. Because high-frequency predictor variables are more likely to form influential predictor variable interactions, we select the important predictor variables by their retention frequency in top triples. We order the retention frequency of every predictor variables in the high-scored triples from high to low. And the first difference of retention frequency sequence usually shows big drops in the beginning and stabilizes around zero later. We choose the cut-off value when the first difference of retention frequency sequence is stable around zero.

Then we get key predictor variables that have high potential to construct important interactions when combined with each other. And the dimension of predictor variables decreases significantly from thousands to hundreds or dozens, which is beneficial for next step.

#### Step 3: Generation of interactions based on BDA

In this step, we realize BDA based on the selected variables in step 2. There are two quantities including the size of the initial subset and the number of repetitions to be determined before applying BDA. The size of the initial subset can be calculated by Poisson approximation [23]. Then the minimum requirement is met if the initial size *k* satisfies

4 $$\frac{n^2}{2m_{k-1}}\ge 1,$$

where n is the number of training samples, and $m_{k-1}$ is the number of partition elements induced by a subset with $k-1$ variables.

The approximation is adequate if the averaged number of observations per partition element is at least 4 [27]. So we can get the lower bound of the initial size. In practice, any initial size between the upper and lower bounds can be used. The number of repetitions in BDA is the number of variable subsets subjected to backward dropping. Assuming *p* is the number of predictor variables, and *k* is the initial size. If we want to cover all interactions with *z* variables ($z\le k$), it is shown that we are expected to have

5 $$\hat{B} \approx \left[ {{{\left( {\begin{array}{*{20}c} p \\ z \\ \end{array} } \right)} \mathord{\left/ {\vphantom {{\left( {\begin{array}{*{20}c} p \\ z \\ \end{array} } \right)} {\left( {\begin{array}{*{20}c} k \\ z \\ \end{array} } \right)}}} \right. \kern-\nulldelimiterspace} {\left( {\begin{array}{*{20}c} k \\ z \\ \end{array} } \right)}}} \right]log\left( {\begin{array}{*{20}c} p \\ z \\ \end{array} } \right).$$

Finally $2{\hat{B}}$ can be used as an upper bound which covers all key predictor variables [23]. Then we get return sets by applying BDA combined with two influence measures separately. And the return sets will undergo two filtering procedures. The first procedure is to filter out return sets with overlapping variables. And we shall keep only one of those return sets containing common variables. This can be done by sorting the return sets in decreasing order according to the influence measure and then removing those having variables in common with a higher-scored one. The return sets after removing overlap ones are subjected to a forward adding algorithm to remove false positives [23]. In order to use interactions selected from the above steps to determine the status of *Y*, we apply the following steps to construct the final classifier.

#### Step 4: Construction of the sub-classifier

We choose Ridge Regression method with cross-validation to construct sub-classifier based on interactions generated above. We include all product terms between variables from one interaction as the joint action of the variables to construct each Ridge Regression sub-classifier. Ridge Regression is appropriate because it alleviates the impact caused by colinearity between predictor variables in one interaction.

#### Step 5: Construction of the final classifier based on Boosting

The sub-classifiers are combined together to construct the final classifier by Boosting algorithm [23], which could greatly improve the performance of the final classifier.

## Results

### Exhibition of experiment results

We state that there are many preprocessing methods for a certain biological dataset, and we choose the best one as far as we know based on step 1. We adopt 5 cross-validation (CV) Experiments in order to evaluate the predictive performance of the model more comprehensively. For every dataset, we divide the samples into five parts. Every time one of five parts is chosen as the test set and the four remaining parts are training set. Then we carry out the whole experiments based on every pair of training and test set. The accuracy is the probability that the predicted category matches the actual category of outcome variable *Y*. The first dataset to be considered is Breast Cancer Wisconsin (Diagnostic) Dataset. Because the dimension of this dataset is small enough, we only need to discretize the data in preprocessing step. After several pre-experiments, we preprocess it by discretizing every predictor variable through all the samples with two-means method. We directly use BDA to find interactions among the predictor variables with $B=14{,}000$ repetitions, the size of initial subset $k =6$ and $z=4$ variables.

Table 2 shows that the average accuracy of MGR method is $93.13\%$ which is better than *I*-score $91.73\%$ on this dataset. Because the number of predictor variables is 30, so we construct Ridge Regression model using all biomarkers without biomarker selection. After selecting key interactions based on MGR, the number of key interactions among 5-CV Experiments is 8, 7, 7, 9, 9 separately. What’s more, every key interaction contains one variable in this example. And the average accuracy by constructing Ridge Regression model based on the corresponding biomarkers is $93.13\%$, which is better than average accuracy $89.80\%$ based on all biomarkers. The results of sensitivity and specificity also explain the advantages of our proposed MGR method. Therefore, we realize dimension reduction after the process of MGR method with improving the accuracy. The second dataset is Leukemia Dataset [20]. The dimension of the dataset is $72\times 3571$ after three procedures applied to the normalized matrix of intensity values (after pooling the 38 mRNA samples from the training set and the 34 mRNA samples from the test set) [22]. Finally, we get the discretized dataset with dimension $72\times 3571$. Details are as follows:

1. Floor of 100 and ceiling of 16,000: the datas are converted to values between 100 and 16,000 after this procedure.
2. Filtering: exclude genes with $max/min\le 5$ or $(max-min)\le 500$, where *max* and *min* refer to the maximum and minimum intensities for a particular gene across the 72 mRNA samples.
3. Base 10 logarithmic transformation.
4. Discretization: we use two-means clustering method for every sample across all genes.

After the preprocessing, the number of genes decreases from 7129 to 3571. It saves eight times of hours with the 3571 genes for step 2 dimension reduction by interacted triples compared with 7129 genes. We conduct 5-CV Experiments on the discretized Leukemia Dataset with dimension $72\times 3571$ based on *I*-score and MGR separately. We choose the parameter $B=1{,}200{,}000$ as the repetition times, $k=8$ as the size of initial subset and $z=4$ variables.

**Table 2:** 5-CV results of Breast Cancer Wisconsin (Diagnostic) Dataset

Method	Exp${\_}$One (%)	Exp${\_}$Two (%)	Exp${\_}$Three (%)	Exp${\_}$Four (%)	Exp${\_}$Five (%)	Average (%)
5-CV results of accuracy						
All biomarkers	${\varvec{84.96}}$	$88.50$	$94.69$	$90.27$	$90.60$	$89.80$
*I*-score	$84.07$	${\varvec{91.15}}$	$93.81$	$95.58$	$94.02$	$91.73$
MGR	$84.07$	${\varvec{91.15}}$	${\varvec{96.46}}$	${\varvec{98.23}}$	${\varvec{95.73}}$	${\varvec{93.13}}$
5-CV results of sensitivity						
All biomarkers	${\varvec{97.78}}$	$96.88$	${\varvec{100.0}}$	$96.39$	$93.41$	$96.89$
*I*-score	$93.33$	${\varvec{98.44}}$	$94.59$	$96.39$	$94.51$	$95.45$
MGR	$93.33$	$96.88$	$98.65$	${\varvec{100.0}}$	${\varvec{96.70 }}$	${\varvec{97.11}}$
5-CV results of specificity						
All biomarkers	$76.47$	$77.55$	$84.62$	$73.33$	$80.77$	$78.55$
*I*-score	${\varvec{77.94 }}$	$81.63$	${\varvec{92.31 }}$	${\varvec{93.33 }}$	${\varvec{92.31}}$	$87.50$
MGR	${\varvec{77.94 }}$	${\varvec{83.67 }}$	${\varvec{92.31}}$	${\varvec{ 93.33 }}$	${\varvec{92.31}}$	${\varvec{87.91}}$

There are two main experiment methods including via biomarker selection and without biomarker selection, where the method via biomarker selection includes *I*-score method and MGR method. For certain method, we conduct 5-CV Experiments. Exp${\_}$One is the first experiment in 5-CV Experiments and so on. Bold data is the best result for every Experiment

**Table 3:** 5-CV results of Leukemia Dataset

Method	Exp${\_}$One (%)	Exp${\_}$Two (%)	Exp${\_}$Three (%)	Exp${\_}$Four (%)	Exp${\_}$Five (%)	Average (%)
5-CV results of accuracy						
*I*-score	$92.86$	$85.71$	$85.71$	${\varvec{100.0}}$	$81.25$	$89.11$
MGR	${\varvec{100.0}}$	${\varvec{100.0}}$	${\varvec{92.86}}$	${\varvec{100.0}}$	${\varvec{93.75}}$	${\varvec{97.32}}$
5-CV results of sensitivity						
*I*-score	$92.86$	$92.31$	${\varvec{100.0}}$	${\varvec{100.0}}$	${\varvec{100.0}}$	$97.03$
MGR	${\varvec{100.0}}$	${\varvec{100.0}}$	${\varvec{100.0}}$	${\varvec{100.0}}$	${\varvec{100.0}}$	${\varvec{100.0}}$
5-CV results of specificity						
*I*-score	${\varvec{100.0}}$	$0$	$80.0$	${\varvec{100.0}}$	$66.67$	$69.33$
MGR	${\varvec{100.0}}$	${\varvec{100.0}}$	${\varvec{90.0}}$	${\varvec{100.0}}$	${\varvec{88.89}}$	${\varvec{95.78}}$

We conduct 5-CV Experiments by using *I*-score method and MGR method on Leukemia Dataset. Exp${\_}$One is the first experiment in 5-CV Experiments and so on. Bold data is the best result for every Experiment

As shown in Table 3, MGR with average accuracy of $97.32\%$ is better than *I*-score with average accuracy of $89.11\%$. The results of sensitivity and specificity also explain the advantages of our proposed MGR method.

### Analysis of experiment results

**Fig. 5:**
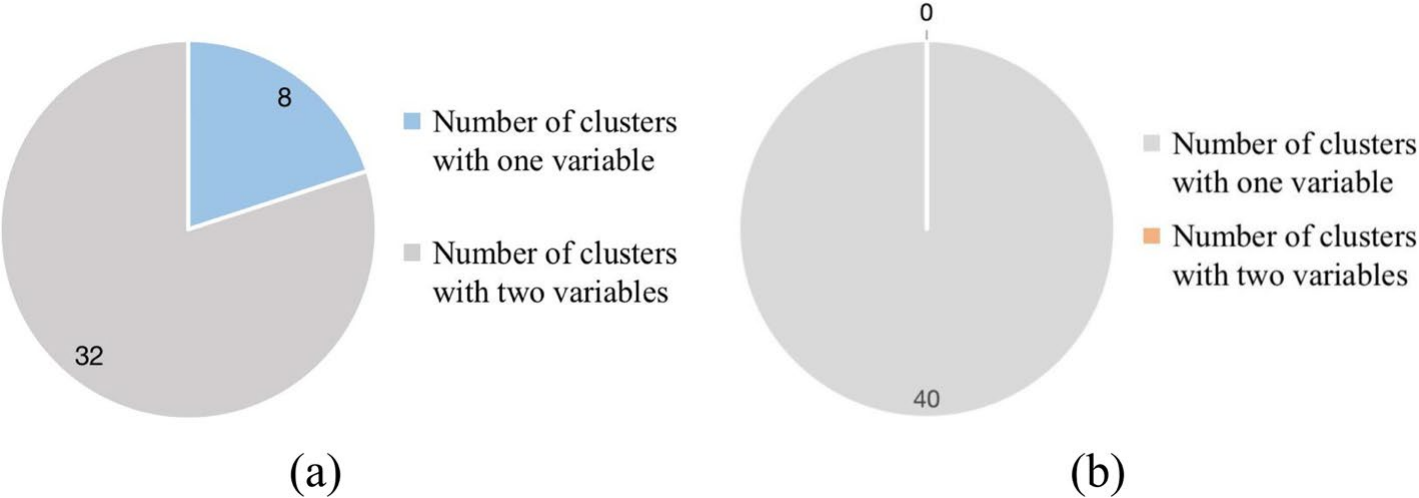
Distributions of interactions selected from Breast Cancer Wisconsin (Diagnostic) Dataset with different number of variables. **a** We get 40 key interactions totally after 5-CV Experiments based on *I*-score, where there are 8 interactions with one predictor variable and 32 interactions with two predictor variables. **b** We get 40 key interactions totally after 5-CV Experiments based on MGR, where every one of the 40 interactions contains one predictor variable

**Fig. 6:**
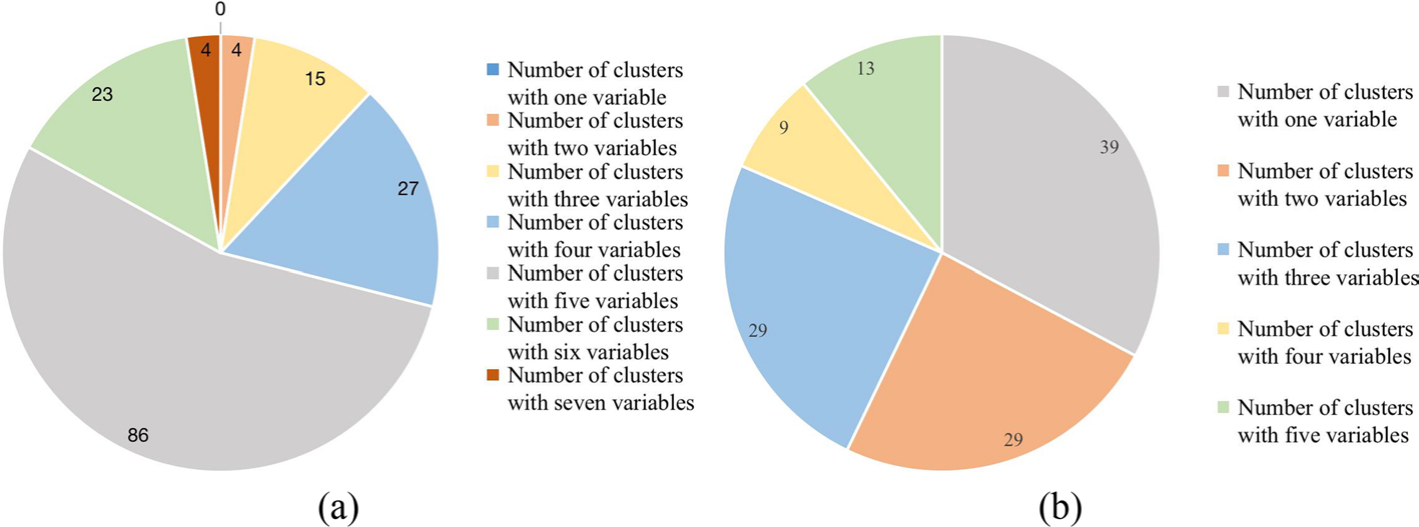
Distributions of interactions selected from Leukemia Dataset with different number of variables. **a** We get 159 key interactions totally after 5-CV Experiments based on *I*-score, where there are 4 interactions with two predictor variables and 15 interactions with three predictor variables until 4 interactions with seven predictor variables. **b** We get 119 key interactions totally after 5-CV Experiments based on MGR and the distribution of the interactions is shown in the plot

In order to figure out the reason MGR behaves better than *I*-score, we need to explore the nature of interactions selected by MGR and *I*-score separately. So we draw diagrams of interactions with different number of variables as shown in Figs. 5 and 6.

Figure 5 displays the distributions of interactions selected from Breast Cancer Wisconsin (Diagnostic) Dataset with different number of variables based on *I*-score and MGR separately. While Fig. 6 displays the distributions of interactions selected from Leukemia Dataset with different number of variables based on *I*-score and MGR separately. From the two diagrams, we can see that *I*-score tends to choose interactions with a large number of variables based on the two datasets. While MGR’s behavior is opposite to it. According to the nature of *I*-score and MGR shown in section methods, MGR is more stable under the circumstance where there are interactions with less variables, which explains why MGR behaves better than *I*-score on the two datasets.

## Discussion

Our proposed influence measure MGR performs better than *I*-score when there are interactions with less number of predictor variables. And the two influence measures can complement each other in real applications. When we get interactions after step 3 based on *I*-score and MGR separately, we can filter these interactions according to the number of predictor variables in them with the properties of *I*-score and MGR. In this paper, we select important interactions by MGR method efficiently. But the internal structure of every interaction is still unknown. A method to explore internal relationship between predictor variables in every interaction is being developed. If the problem is solved, the mechanisms of how biomarkers affect disease will be clearer. Another challenge is that if there are tens of thousands of variables or even larger, computing power will limit the use of the proposed algorithm. Because the number of variables in key interaction is generally not very large in reality, the problem can be solved by finding the method of preliminarily screening possible variables for constituting an interaction together. In other words, we group predictor variables in advance according to their correlation with each other. We will continue to explore and solve these problems.

## Conclusion

It has been a long-lasting interest in the bioinformatics field for detecting biomarker interactions. In this paper, we propose MGR which is proved to be an effective influence measure for selecting interaction between biomarkers. Compared to existing measure *I*-score, we illustrate that MGR behaves better on the circumstance where there are interactions with less variables. By carrying out experiments on two real datasets, the MGR results are better than *I*-score which proves the effectiveness of MGR method. Our proposed MGR is a flexible indicator that can be used when combined with other algorithm structure. In addition to the two kinds of datasets in the paper, MGR method can be applied to other kinds of real biomarker datasets for selecting interactions such as SNPs datasets and protein datasets. We believe that the proposed MGR method can be a useful tool in the area of biotherapies.

## Data Availability

Operating system(s): Windows. Programming language: C and Python. The Breast Cancer Wisconsin (Diagnostic) Dataset analysed during the current study is available in the University of California Irvine Machine Learning repository, [http://archive.ics.uci.edu/ml/machine-learning-databases/breast-cancer-wisconsin]. The Leukemia Dataset analysed in this work can be downloaded in Kaggle repository, [https://www.kaggle.com/crawford/gene-expression].

## Abbreviations

MGR: Multivariate gain ratio
GR: Gain ratio
BDA: Backward dropping algorithm
CV: Cross-validation
